# Precise detection of genomic imbalances at single-cell resolution reveals intra-patient heterogeneity in Hodgkin’s lymphoma

**DOI:** 10.1038/s41408-019-0256-y

**Published:** 2019-11-21

**Authors:** Chiara Mangano, Alberto Ferrarini, Claudio Forcato, Marianna Garonzi, Paola Tononi, Rossana Lanzellotto, Andrea Raspadori, Chiara Bolognesi, Genny Buson, Gianni Medoro, Michael Hummel, Francesca Fontana, Nicolò Manaresi

**Affiliations:** 1 Menarini Silicon Biosystems S.p.A, Bologna, Italy; 2 Charité - Universitätsmedizin Berlin, Institut für Pathologie, Berlin, Germany

**Keywords:** Hodgkin lymphoma, Cancer genomics

Dear Editor,

Classical Hodgkin lymphoma (cHL) is a B-cell-derived lymphoproliferative disorder^1^ characterized by the presence of morphologically characteristic pathognomonic malignant cells termed Hodgkin and Reed–Sternberg (HRS) cells. HRS cells are derived from germinal center B cells and represent only a small fraction (usually <5%) of all the cells in the tumor tissue, whereas the surrounding inflammatory milieu is rich in T cells, B cells, granulocytes, eosinophils, macrophages, and stromal cells^2^.

Despite the extensive inflammatory microenvironment, HRS cells are able to escape immune surveillance using several mechanisms, including overexpression of programmed cell death protein-1 (PD-1) ligands (PD-Ls), upregulated in a dose-dependent manner by copy-number alterations (CNAs) of chromosome 9p24.1, a locus that encodes PD-L1/PD-L2 as well as Janus kinase 2 (JAK2), which further enhances PD-L expression through the JAK2/STAT (signal transducer and activator of transcription) pathway. PD-L locus amplification has been associated with advanced stages of the disease and with a shorter progression-free survival^3,4^. Along with PD-L locus copy alterations, HRS cells display a general abnormal karyotype, including gains and losses extended evenly to whole chromosomes^3^. Genetic alterations of HRS cells are a valuable source of information to develop new treatments or predictive/prognostic biomarkers. However, rareness of HRS cells dispersed among the surrounding inflammatory milieu poses technical challenges to unravel malignant cells’ genetic alterations.

To enable isolation and molecular characterization of HRSs without interference from nonmalignant cells, we set up an optimized workflow to isolate single HRSs starting from formalin-fixed paraffin-embedded (FFPE) tissue biopsies of cHL patients by disaggregation to a single-cell suspension and staining using membrane protein CD30 for HRS cell identification (for protocol details see Supplementary Methods and Supplementary Figs. 1–3).

To isolate single HRS cells, we leveraged on DEPArray™, an image-based cell-sorting technology, previously successfully applied for single-cell isolation in liquid biopsy^5,6^ and for characterization of pools of cells from FFPE tissues^7^. In addition, we carried out single FFPE cell genomic profiling, based on *Ampli*1™ WGA and *Ampli*1™ LowPass, previously applied to single-cell liquid biopsy^5,6^. The proposed method shows as a proof of concept that it is possible to identify genome-wide CNAs at the single-cell level and obtain information about inter-tumor and intra-tumor heterogeneity.

The immunofluorescent pattern obtained for HRS cell identification is shown in Supplementary Fig. 4: CD30 signal is clearly localized in the cytoplasmic/membrane compartment and allows discriminating CD30-positive HRS cells from CD30-negative cells.

DEPArray™ digital sorting, a highly automated image-based platform, allowed us to identify and recover whole pure HRS cells combining multiple marker expression and localization and cell morphology. Indeed, HRS cells have generally a polyploid genome/multinuclear appearance, and consequently, a higher DNA content compared with leukocytes. Thus, DAPI (4′,6-diamidino-2-phenylindole) signal intensity and cell/nucleus morphology were employed, in combination with CD30 signal intensity, to discriminate between small and rounded diploid leukocyte cells, with no or basal CD30 expression, and larger hyperdiploid HRS cells showing higher CD30 expression (Fig. 1a–c).

**Fig. 1:**
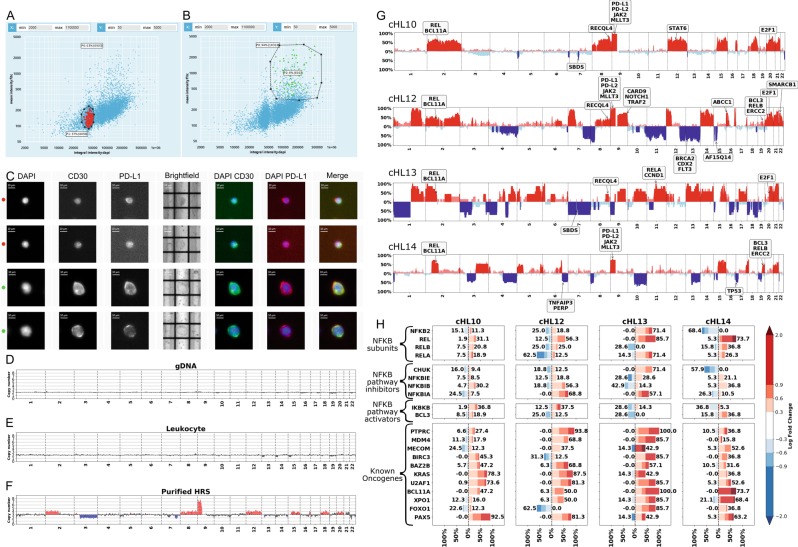
Detection of genomic imbalances in single HRS cell selected by DEPArray™ digital sorting technology. Scatter plots of CD30 mean intensity (*y*-axis) versus integral intensity DAPI (*x*-axis) of **a** leukocytes and **b** HRS cells. Selected leukocytes and HRS cells are highlighted in red and green, respectively. **c** Image gallery of two representative leukocytes and two representative HRS cells (labeled with red and green dots, respectively). **e**, **f** Representative whole-genome copy-number profiles of a single leukocyte and a single HRS cell confirm the nonmalignant and tumor nature of the cells, respectively. **d** Representative whole-genome copy-number profile obtained from the analysis of purified bulk DNA from the same patient, showing, as expected, no alterations given the rareness of HRS cells and the corresponding high contamination from normal cells. **g** Distribution of copy-number gains (red) and losses (blue) across all single HRS cells of each patient. An absolute log-fold change of at least 0.3 was used as threshold. *Y*-axis represents the percentage of single cells showing the CNAs. CNAs that were present in more than 30% of all single cells for each patient are highlighted in a darker color shade. Labels show genes in conserved cHL-related alterations. Recurring copy-number imbalances were observed with the most frequent gains on 9q, 2p, 8q, and 20q. These regions include genes encoding proteins associated with immune response (PD-L1, PD-L2), JAK-STAT signaling (*JAK2*), *MLLT3*, which is a component of the super elongation complex (SEC), *REL* (NF-κB pathway), *BCL11A*, belonging to a highly recurrent minimal region of gain in cHL, *RECQL4* gene, a DNA helicase that belongs to the RecQ helicase family and has been previously been associated with predisposition to an increased risk in developing cancer, and E2F-1, a transcription factor that, in Hodgkin lymphoma, is upregulated and has been related to tumor kinetics. **h** Percentage of altered HRS cells for key components of NF-κB pathway and known oncogenes. Different levels of copy-number change, expressed as log-fold change, are highlighted in different color shades. Genome-wide analysis at single-cell resolution allowed us to observe a high degree of heterogeneity in terms of copy-number alteration level for genes in the NF-κB pathway both between and within samples. Strikingly, while *REL* showed copy-number gains in all four patients, it was only moderately gained (0.6 < log FC ≤ 0.9) in two out of four patients, while in the fourth patient (cHL14) *REL* showed large increases in copy number (log FC > 2).

Furthermore, as PD-L1 is often overexpressed in HRS cells, its staining signal intensity was analyzed for all selected HRS cells and compared with the respective leukocyte population showing a much higher signal intensity in HRS cells than in leukocytes, in each analyzed patient (Supplementary Fig. 5).

A total of 213 CD30+ cell recoveries, 27.7% of which had rosetting T cells (Supplementary Fig. 6), and 87 CD30− cell recoveries were performed from tissue samples collected from four cHL patients (Supplementary Table 1). Following filtering (Supplementary Methods and Supplementary Figs. 7–9), genome-wide copy-number profiles of 148 CD30+ cells and of 87 CD30− putative leukocytes have been successfully obtained by low-pass whole-genome sequencing. Library success rate was highly related to FFPE tissue quality (Supplementary Fig. 10). Most CD30+ cells (98.0%) showed genomic alterations, while all CD30− cells showed a flat profile, confirming that the selection employed was effective in isolating aberrant cells (Fig. 1e, f). Figure 1d shows a representative whole-genome copy-number profile obtained from analysis of purified bulk DNA from the same patient, showing, as expected, no alterations given the rareness of HRS cells and high contamination from normal cells.

Out of 148 CD30+ single cells, 79.7% had large CNA calls (>20 Mbp; *P* value < 0.01), and on average, 36.2 segments (11.0 copy-number loss segments; 25.2 copy-number gain segments) were identified per sample accounting for 27.0% of the genome with values ranging from 1% up to 57.5% of genome. Altered segment lengths ranged from 78 kbp to 243 Mbp (average = 32.5 Mbp) for gains and from 1 to 200 Mbp (average = 27.9 Mbp) for losses. Among altered regions, a preponderance of gains was observed, contributing, on average, to 70% of aberrant genome.

Figure 1g shows the composite profiles summarizing the imbalances affecting the autosomes across all single cells analyzed for each patient. Some of the detected CNAs were conserved across patients and patient’s single cells, while others were present only on a subset of samples. Among conserved ones, CNAs were detected in regions known to harbor recurrent gains (chromosomes 2p, 8q, and 9q) and losses in cHL^8^. These regions contain genes of pathways known to be altered in cHL, like REL/NF-κB (nuclear factor-κB) and JAK/STAT pathways, which may be involved in the constitutive activation of proliferative and antiapoptotic phenotype of HRS cells^9^. The constitutively activated NF-κB pathway in HRS has been extensively reported and has been related to copy-number gains of genomic regions containing positive regulators like *REL* and *MAP3K14*^9,10^.

One patient displayed a high-level amplification of *REL* gene together with the copy-number loss of NF-κB inhibitor *CHUK*^9^ (Fig. 1h). CNAs in regions adjacent to *REL* comprised the *FOXO1* gene (RNA translocation) and regulatory proteins, including p53, which has been reported to be deregulated in cHL. In the same region, we detected alterations ranging from few copy gains to amplification of *BCL11A*^11^, which has been shown to be involved in lymphomas pathogenesis^9^.

High levels of genetic imbalances were detected in several oncogenes, already reported as altered in cHL (*MDM4* and *U2AF1*), and in other lymphoproliferative malignancies.

Finally, the region containing *PD-L1*/*PD-L2*/*JAK2* showed gains in most malignant cells of three patients. Interestingly, these alterations displayed highly variable copy-number levels between different HRS cells of the same patient, ranging from polysomy to amplifications, in agreement with a previous report showing a high heterogeneity of *PD-L1* copy-number levels, across different cells in the same patient^3,4,12^ (Fig. 2a, b). Noteworthy, one patient displayed, in almost all analyzed HRS, a copy-number loss of PD-L region, which has not been reported before. However, the same patient presented a highly altered genomic profile, with whole-chromosome gains and losses, and the loss of PD-L region may be a consequence of a massive chromosomal instability. Recently, the use of PD-1 blockade has been demonstrated as an effective therapeutic regimen in cHL patients with relapsed/refractory disease^12,13^, and PD-1 blockade effectiveness has been correlated to PD-L locus amplification detected using fluorescence in situ hybridization^3,12^, a low-throughput and labor-intensive technology, with a limited number of probes multiplexed in a single experiment. Next-generation sequencing-based absolute copy-number profiling of single HRS may provide a valuable tool to uncover alterations at genome-wide level that can be useful for predicting outcome of a specific treatment regimen, for example, for the adoption of immune therapy.

**Fig. 2:**
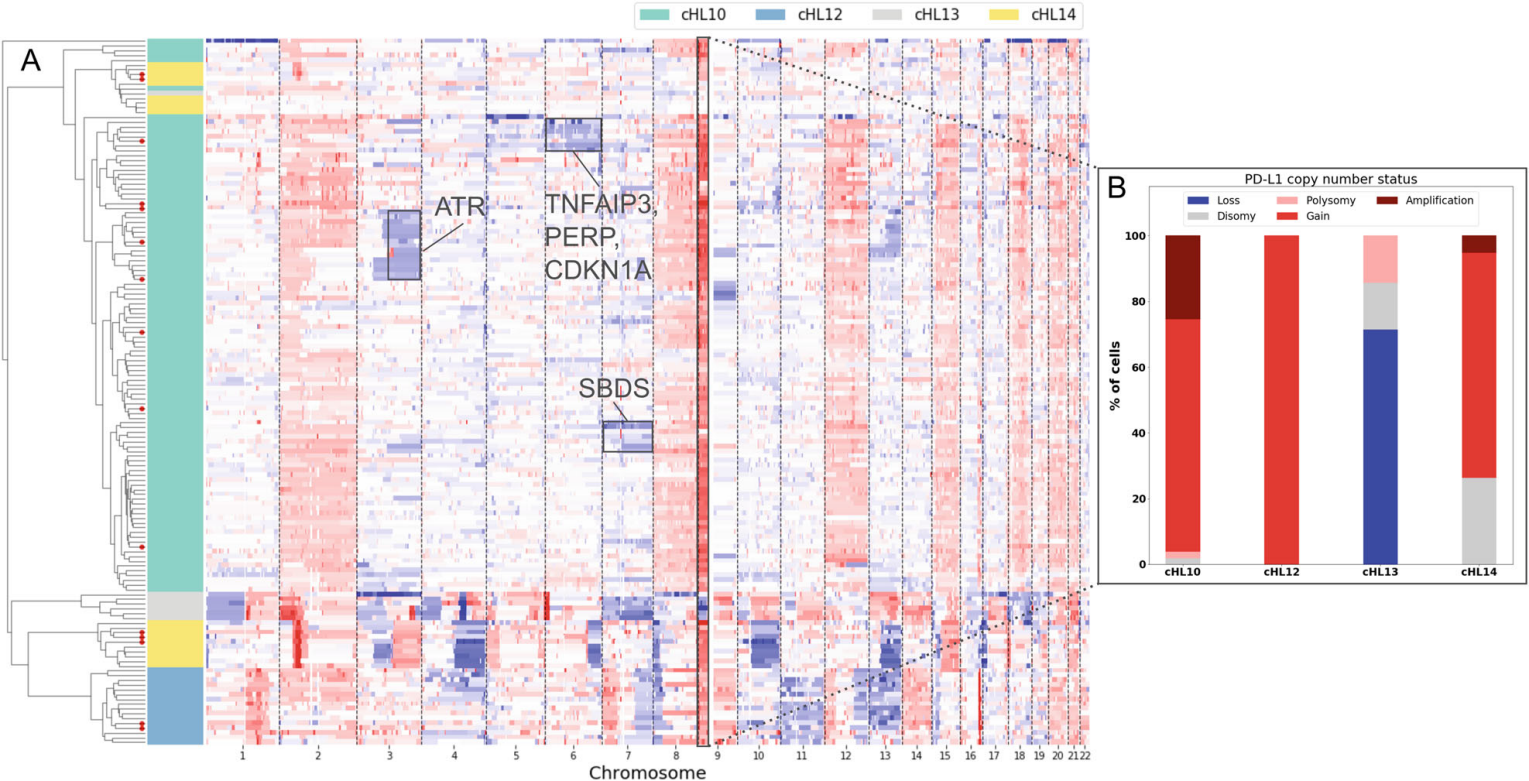
Intra-patient copy-number profile heterogeneity. **a** Unsupervised hierarchical clustering of genome-wide copy profiles of 148 purified HRS cells. Hierarchical clustering of copy-number profiles, expressed as log-fold change (base = 2), was performed using correlation metric and average method. HRS cells of the four different patients (cHL10, cHL12, cHL13, and cHL14) are labeled by the left-side colored bar. In the heatmap, each row represents the copy-number profile of a single cell along the genome (*x*-axis). Copy-number gains and losses, with respect to the main ploidy, are shown in red and blue, respectively. HRS cells rosetted by the surrounding leukocytes are highlighted with red dots on the dendrogram. Profiles of rosetted HRS cells were adjusted taking into account the fraction of the nonmalignant component in each sample. The arrow highlights a cluster formed from three single-cell profiles showing no alterations. Genome-wide copy-number profiles highlight several regions showing heterogeneity in terms of the presence/absence of CNAs among different subpopulations in the same patient (black boxes). In particular, subclones with copy-number losses in 3q, chromosome 6, and chromosome 7 were identified among single cells from individual cHL10. These regions include genes such as *ATR*, involved in DNA repair, *TNFAIP3*, which is a tumor-suppressor gene in HL, *PERP*, the p53 apoptosis effector, and the tumor suppressor *CDKN1A*, codifying the protein p21, which promotes cell cycle arrest in response to many stimuli and functions both as a sensor and as an effector of multiple antiproliferative signals. **b** PD-L1 copy-number status across different single cells in the four cHL patients shows a high degree of copy-number heterogeneity both across different individuals and between different single cells in the same individual for *PD-L1*. PD-L1 copy-number status was classified as gain when the fold change, with respect to genome main ploidy, was between 1 and 3, as the fold change of amplification was ≥3.

The ability to characterize copy-number profiles at single-cell resolution allowed to zoom in on single patients revealing subclonal copy-number events. In particular, patient cHL10, for which 106 HRS single cells were individually characterized, showed copy-number loss regions in chromosomes 3, 6, or 7, constituting about 13%, 5%, and 7% of the total of patient cells analyzed, respectively (Fig. 2a, black boxes). Noteworthy, most clonal alterations detected were gains in highly recurrent genomic locations containing driver genes such as *PD-L1/PD-L2* and *REL*, while subclonal alterations in examined cases were mainly losses in regions containing tumor suppressors (*TNFAIP3* and *CDKN1A*) and genes associated with DNA repair (*ATR*). *ATR* has already been proposed to have a role in lymphomagenesis in HL as deletions and insertions in the gene have been shown to cause a delay/abrogation in double-strand break and single-strand break repair and defects in the accumulation of p53^14^. Late mutations in cancer genes involved in maintenance of genome integrity through DNA damage response and repair have already been observed also in other cancer types, and it has been proposed that such mutations may provide advantages to emerging subclones later in evolution^15^.

Various techniques have been employed to overcome the hurdle of assessing HRS cells’ genetic alteration in cHL patients due to the rareness of target tumor cells. However, the approaches proposed up to now only achieved a partial enrichment of tumor component and did not allow to disentangle subclonal heterogeneity, which may be important for tumor evolution and development, as recently suggested by studies in other tumor types^15^. The workflow presented in this paper addresses the challenges of genetic characterization caused by low tumor cellularity, allowing, for the first time in cHL, to perform genome-wide copy-number profiling at the single-cell level.

Finally, we feel that it will be important in the future to perform additional studies to assess the extent of heterogeneity across larger patient cohorts and to identify potentially clinically significant biomarkers for tumor evolution and response to therapeutic regimens.

## Supplementary information

Supplementary information
