# Supplementary material for: Precise detection of genomic imbalances at single-cell resolution reveals intra-patient heterogeneity in Hodgkin’s lymphoma
Source: Blood Cancer J. 2019 Nov 21;9(12):92. doi: 10.1038/s41408-019-0256-y (PMC6872566; doi:10.1038/s41408-019-0256-y)
Supplement: Supplementary file 1 — Supplementary information [file 41408_2019_256_MOESM1_ESM.pdf]

Supplementary information

Precise detection of genomic imbalances at single cell resolution reveals intra-patient heterogeneity in Hodgkin's lymphoma

Authors

Chiara Mangano[1,*], Alberto Ferrarini[1,*], Claudio Forcato[1], Marianna Garonzi[1], Paola Tononi[1], Rossana Lanzellotto[1], Andrea Raspadori[1], Chiara Bolognesi[1], Genny Buson[1], Gianni Medoro[1], Michael Hummel[2], Francesca Fontana[1], Nicolò Manaresi[1]

Authors affiliations

1 Menarini Silicon Biosystems S.p.A., Bologna

2 Charité - Universitätsmedizin Berlin, Institut für Pathologie, Berlin

* these authors contributed equally to this work

SUPPLEMENTARY METHODS

Tissue specimens

Tissue sections (50 µm) from formalin-fixed paraffin-embedded (FFPE) lymph node and spleen biopsies, from n=4 classical Hodgkin lymphoma patients, were obtained from Discovery Life Sciences biobank (Supplementary Table 1).

FFPE tissue section dissociation, immunofluorescent staining of cell suspension

FFPE tissue sections were dewaxed and disaggregated into cell suspensions following a modified protocol based on previously described procedures[1,2].

One section with a thickness of 50 µm was cut from a FFPE tissue block and put on the bottom of a GentleMACS™ C Tube (Miltenyi Biotec, Germany). FFPE section was dewaxed by three 10 min sequential incubations in Bio Clear (Bio-Optica, Italy) and then rehydrated by sequential 1 min incubations in 100% ethanol (three times), 70% ethanol (three times), 50% ethanol (two times). The hydration process was completed with 1 min incubations in deionized water.

For Heat-Induced Antigen Retrieval (HIAR), the section was pre-incubated in 100mM Tris base and 10mM EDTA solution (pH 8.0) for 1 min at room temperature and heat-treated in the same pre-warmed buffer for 30 min at 95°C. After cooling down at room temperature, the section was washed with HBSS medium (Thermo Fisher Scientific, MA). In order to obtain a cell suspension, the section was incubated in 5 ml of 0.15% collagenase IV

(Thermo Fisher Scientific, MA) 5mM calcium chloride solution (Merck KGaA, Germany) in HBSS medium, at 37 °C in a water bath. Every 10 minutes of incubation, the samples were subjected to mechanical dissociation using Gentle MACS Dissociator (Miltenyi Biotec, Germany). The dissociation process was monitored using a microscope and the procedure was repeated for a maximum of 40 minutes or until all the tissue pieces disappeared (Supplementary Figure 2). The dissociation process was stopped by putting the tube on ice.

The solution containing cell suspension was resuspended and transferred through a 100 µm mesh nylon filter into a 15 ml conical tube. The C Tube was washed with 5 ml of ice-cold washing buffer (0.6% BSA, 16 mM Tris, 0.12M NaCl, 0.04% Tween-20, pH 7.5) that was then transferred through the same filter.

Cell suspension was centrifuged 5 min at 1,000 g, to spin down the cells and the pellet was washed two times with ice-cold washing buffer.

An aliquot of $5x10^5$ cells was incubated for 30 minutes at RT in hydrogen peroxide 0,15% diluted in PBS to quench the endogenous peroxidase. Cells were washed in washing buffer, centrifuged and incubated overnight at +2…+4°C with of goat serum 10% solution to block the non-specific binding. Cells were labelled in a premixed solution of PD-L1 primary antibody (Ventana SP263, cat.790-4905, Roche, Switzerland) and 3 µg/mL of CD30 primary antibody (BerH2, cat.M075101-2, Agilent Technologies, CA) for 1 hour at RT.

Cells were washed twice with washing buffer, centrifuged at 1,000 × g for 5 min, and then incubated for 30 minutes at RT in the dark with secondary antibody Alexa Fluor® 546 Goat Anti-Rabbit (cat.A-11035, Thermo Fisher Scientific, MA) for PD-L1 detection and DAPI. After incubation, the sample was washed twice with washing buffer, centrifuged at 1,000 × g for 5 min, and incubated for 30 minutes at RT with Reagent 1 (Mouse Antibody Enhancer) provided in Polink-2 HRP Plus Mouse DAB Detection System for Immunohistochemistry (GBI Labs, WA). Cells were washed twice with washing buffer, centrifuged at 1,000 × g for 5 min, and then incubated for 30 minutes at RT with Reagent 2 (POLYMER-HRP for Mouse) of Polink-2 HRP Plus Mouse DAB Detection System for Immunohistochemistry. The sample was then washed with 1 ml of PBS and incubated for

10 minutes at RT in a premixed solution containing 1 µl of Alexa Fluor™ 488 Tyramide Reagent (Thermo Fisher Scientific, MA) in 99 µl of Reagent 3A (DAB Substrate) of Polink-2 HRP Plus Mouse DAB Detection System for Immunohistochemistry for CD30 detection. After the 10 minutes, HRP reaction was stopped adding 10 mM EDTA and washing with 1 ml of washing buffer. The cell pellet was resuspended in 200 µl of ice-cold washing buffer.

Method set-up

To limit damages to target membrane proteins during the dissociation process, collagenase IV was used as it has a low tryptic activity and it is especially gentle with membrane antigens. Furthermore, the time of exposure to the enzyme was reduced to the minimum, monitoring the dissociation by repeated microscope observation of sample aliquots (Supplementary Figure 2). The final procedure was the result of numerous tests carried on both tissue sections on microscope slides and on dissociated cell suspension (Supplementary Figure 3). Indirect immunohistochemistry (IHC) using a fluorescent dye-conjugated secondary antibody for detection was compared to immunoenzymatic assays designed to amplify the signal catalyzed by the enzyme horseradish peroxidase (HRP) (Supplementary Figure 3A, B and C). While none or a very weak signal was obtained using indirect immunohistochemistry, especially on dissociated sample (Supplementary Figure 3D and G), using the enzyme-based Tyramide Signal Amplification (TSA) technology it was possible to detect positive cells (Supplementary Figure 3E and H). To maximize the detection of the non-degraded fraction of CD30 antigen, DAB (3,3′-Diaminobenzidine) Detection System was used to boost the signal. Using DAB technique, it was possible to identify numerous HRS cells expressing a clear and strong signal (Supplementary Figure 3F and I). The combination with tyramide reagent as HRP substrate allowed us to obtain an amplified fluorescence emission detectable in the selected fluorescent channel.

DNA quality control

A volume corresponding to 1.500 stained cells was transferred into a 0,2ml tube containing PBS.

Cells were centrifuged at 14.100g for 10 minutes and the PBS was gently removed following the liquid meniscus during the aspiration. Cells were then lysed using DEPArray™ LysePrep Kit (Menarini Silicon Biosystems) according to manufacturer instructions. The quality of DNA was determined using the DEPArray™ FFPE QC Kit (Menarini Silicon Biosystems), a qPCR-based assay yielding a QC score using two different primer pairs that produce amplicons of 54 bp and 132 bp (see Supplementary Figure 10)

gDNA Extraction

Genomic DNA was purified from one 50 µm section of FFPE lymph node and spleen biopsies using QIAamp DNA FFPE Tissue Kit (QIAGEN, Germany).

Detection, isolation and recovery of HRS cells by DEPArray™ sorting technology

A small amount of the labeled cell suspension was washed 16 to 72 hours before the DEPArray™ run, with 1 ml of SB115 buffer (Menarini Silicon Biosystems, Italy) and then twice just before the DEPArray™ run by centrifugation at 1000g for 5 min. Pellet was resuspended in the same buffer to obtain 1800-2000 cells/µl. DEPArray™ NxT cartridge (Menarini Silicon Biosystems, Italy) was loaded with 2.5 ml of SB115 buffer and 12 µl of sample.

After loading the labeled single cell suspension in the cartridge, cells are injected into the main chamber of the chip, randomly distributed and trapped in stable levitation in the nearest DEP cages. The fluorescent

microscope integrated in the DEPArray™ NxT instrument allows the acquisition of high-resolution real time images in the selected fluorescent channels. Chip Scan is carried out selecting the following channels: Brightfield, DAPI, FITC (for CD30-), PE (for PD-L1-). All the events are identified automatically using DAPI as common marker. Qualitative and quantitative marker evaluation, along with cell DNA content measurement, was performed with the CellBrowser™ analysis software integrated in DEPArray™ NxT system. The leukocytes population is used as an internal DNA-diploid reference for DNA ploidy, the population with lower integral intensity DAPI was gated to identify leukocytes population, while CD30 positive hyperdiploid population was gated to identify HRS cells. Selected cells were then recovered individually either in single tubes or in 96 well plates.

While the DEPArray can select up to 1,000 cells (up to 96 single-cell recoveries and/or pools of up to 507 cells) the actual output was limited by the number of HRS cells present in the loaded sample. This is dependent on the percentage of tumor cells in the lymph node and on their density in the biopsied area. This aspect goes beyond the scope of this work, but we foresee that one approach to maximize the number of HRS recovered may be to dissect the tissue area with the higher presence of HRS prior to dissociation.

Sample Preparation for downstream Analysis

In case of single tube recoveries, to reduce the collected volume, tubes containing recovered cells in SB115 buffer were centrifuged at 14,100 g for 10 min in a fixed rotor centrifuge. 50 µl of buffer were removed, 100 µl of PBS were added and tubes were centrifuged at 14,100 g for 10 min in a fixed rotor centrifuge. All buffer volume was aspirated. In case of 96 well plate recoveries, 100 µl of PBS were dispensed in each well containing cell recoveries and the plate was centrifuged at 2000xg for 40 minutes at room temperature. A customized protocol was developed and implemented on STARLet liquid handler (Hamilton Life Sciences, Italy) to reduce the collected volume.

Whole genome amplification and NGS library preparation

DNA of isolated cells was amplified using the *Ampli*1™ WGA kit[3,4] (Menarini Silicon Biosystems, Italy) according to manufacturer instructions. Quality of Ampli1™ WGA products was checked using Ampli1™ QC kit (Menarini Silicon Biosystems). However, WGA GII was not predictive of final library quality and is therefore not suitable as quality control for single FFPE cells (see Supplementary Figure 10B).

An aliquot of 5 µl of *Ampli*1™ WGA product was transferred into a new tube and DNA libraries were prepared using either Ampli1™ LowPass Kit for Illumina® or Ampli1™ LowPass Kit for Ion Torrent™ (Menarini Silicon Biosystems, Italy)[5].

For Illumina compatible libraries, quantification by qPCR and pooling were performed according to Ampli1™ LowPass Kit for Illumina® (Menarini Silicon Biosytems, Italy) instructions for use. Single-end sequencing was performed on MiSeq® Instrument using *Ampli*1 SEQ custom primer, supplied with Ampli1™ LowPass Kit for Illumina®, and MiSeq Reagent Kit v3 (150 cycles; Illumina®, CA).

In case of 96 well plate recoveries, a customized protocol was developed and implemented on STARLet liquid handler (Hamilton Life Sciences, Italy) to amplify isolated purified cells genomic DNA with Ampli1™ WGA and to generate *Ampli*1™ LowPass libraries.

For Ion Torrent compatible libraries, the library pool was used for emulsion PCR amplification (400bp) and template-positive Ion Sphere Particles (ISPs) were enriched using the Ion Chef™ System (Thermo Fisher Scientific, MA). Sequencing was performed using Ion 530™ chip on Ion S5™ System (525 flows).

Bioinformatic data analysis

Raw reads, produced by Illumina® MiSeq® sequencer or Ion S5™ System, were analyzed with MSBiosuite bioinformatics platform (Menarini Silicon Biosystems, Italy). In brief, raw reads were aligned to reference

genome (hg19) using BWA (v0.7.12) with MEM algorithm and Ion Torrent Suite (v5.8.0), respectively. Alignments with quality score > 5 were retained and processed with Control-FREEC (v11.0)[6] using the control-free mode and coefficientOfVariantion parameter set to 0.06.

To correct profiles for contamination from leukocytes ContaminationAdjustment parameter was set as follows:

$$contaminationAdjustment = \frac{number\ of\ leukocytes}{total\ number\ of\ cells}$$

Multiple profiles were generated for each sample by setting the ploidy parameter to values in the range from 2 to 6 and were evaluated and a profile was selected as best fitting if it showed the lowest Residual Sum of Squares (RSS) and the highest percentage of genome explained.

Alignments were randomly subsampled to 200,000 aligned reads and copy number analysis was performed with Control-FREEC (v11.0) using a window length = 2 Mbp. Derivative Log Ratio Spread (DLRS) was then calculated:

$$DLRS = \frac{std(diff(FC))}{\sqrt[2]{2}}$$

where *std* is the standard deviation of *diff*, the n-th discrete fold change (FC) difference between consecutive non-overlapping windows.

To estimate the level of diversity of the libraries a score, named R50, has been calculated:

$R50 = \frac{N_{100,000}}{N_{200,000}} x100$, where:

- $N_{100,000}$ = number of Ampli1 fragments, sorted from most covered, covered by a total of 100,000 reads.
- $N_{200,000}$ = number of fragments covered by a total of 200,000 reads.

Final dataset was obtained by filtering a total of 300 pure cells copy number profiles generated from n=4 patients (cHL10: n=166; cHL12: n=57; cHL13: n=45; cHL14: n=32), two thirds of which corresponded to CD30+ putative tumor cells and the remaining one third were CD30- cells.

About 30 % of CD30+ selected cells still retained rosetting leukocytes after dissociation: this is in line with several studies describing the presence of rosetting T cells trying to eradicate the disease but failing to recognize and eliminate tumor cells due to several immune evasion mechanisms[7,8] (Supplementary Figure 6A). Nonethless, thanks to the image-based selection adopted in this workflow, it is possible to preferentially select single HRS cells or, in order to increase the number of recoveries, to count the number of “contaminant” leucocytes for each rosetted cell. Such information is used as input in the bioinformatic data analysis (see Bioinformatic data analysis section) in order to correct the signal for contaminant cells (Supplementary Figure 6B,C).

The majority of CD30+ recoveries analyzed (72.3%) were single cells or had up to 1 contaminant leucocyte (88.3%; Supplementary Figure 6). A total of 12 CD30+ single cell profiles were excluded because of an almost flat profile accompanied by a low R50, indicating a low library complexity. Further 2 CD30+ single cell profiles were excluded because of small cell size associated with a flat profile, indicating that they were CD30 activated leukocytes or, less likely, tumor cells displaying a rather normal copy number profile. Additional 18 single cell-recoveries were filtered because of noisy profiles which did not cluster with the high quality ones (DLRS < 0.4) in the first 2 components PCA space (Supplementary Figure 7). Finally, 33 single cells contaminated by leukocytes, were removed because the profiles did not cluster or were dissimilar from any other profile obtained from the same patient (Supplementary Figure 8). Most of the profiles from single pure CD30+ cells passed filtering steps (86%) and about one third of those obtained from 34 single CD30+ cell contaminated from a single leukocyte could be retained after filtering (Supplementary Figure 9).

Availability of data and materials

The datasets generated and/or analyzed during the current study are available in the Open Science Framework repository, osf.io/xyh6g

Code availability

Code to generate figures contained in this paper is available upon request.

References

1 Bolognesi C *et al.* Digital Sorting of Pure Cell Populations Enables Unambiguous Genetic Analysis of Heterogeneous Formalin-Fixed Paraffin-Embedded Tumors by Next Generation Sequencing. *Sci Rep* 2016; **6**: 20944.

2 Corver WE, ter Haar NT. High-Resolution Multiparameter DNA Flow Cytometry for the Detection and Sorting of Tumor and Stromal Subpopulations from Paraffin-Embedded Tissues. In: *Current Protocols in Cytometry*. John Wiley & Sons, Inc.: Hoboken, NJ, USA, 2011, pp 1–21.

3 Arneson N *et al.* Comparison of Whole Genome Amplification Methods for Analysis of DNA Extracted from Microdissected Early Breast Lesions in Formalin-Fixed Paraffin-Embedded Tissue. *ISRN Oncol* 2012; **2012**: 1–10.

4 Stoecklein NH *et al.* SCOMP is superior to degenerated oligonucleotide primed-polymerase chain reaction for global amplification of minute amounts of DNA from microdissected archival tissue samples. *Am J Pathol* 2002; **161**: 43–51.

5 Ferrarini A *et al.* A streamlined workflow for single-cells genome-wide copy-number profiling by low-pass sequencing of LM-PCR whole-genome amplification products. *PLoS One* 2018; **13**: e0193689.

6 Boeva V *et al.* Control-FREEC: A tool for assessing copy number and allelic content using next-generation sequencing data. *Bioinformatics* 2012; **28**: 423–425.

7 Fromm JR, Kussick SJ, Wood BL. Identification and Purification of Classical Hodgkin Cells From Lymph Nodes by Flow Cytometry and Flow Cytometric Cell Sorting. *Am J Clin Pathol* 2006; **126**: 764–780.

8 Carey CD *et al.* Topological analysis reveals a PD-L1-associated microenvironmental niche for Reed-Sternberg cells in Hodgkin lymphoma. *Blood* 2017; **130**: 2420–2430.

Supplementary Tables

Patient ID	Age	Gender	Ethnicity	Date of Procurement	Harvest Site	Tumor Tissue Type (primary or metastatic)	Race	Histological type	Overall Treatment Status	Stage
cHL10	80	Male	Unknown	01/01/2016	Lymph Node	Unknown	Unknown	Nodular Sclerosing	Unknown	Unknown
cHL12	84	Male	Caucasian	05/11/2003	Lymph Node	Primary	White	Nodular Sclerosing	Pre Treatment	Unknown
cHL13	56	Male	Caucasian	02/07/2004	Lymph Node	Primary	White	Nodular Sclerosing	Pre Treatment	Unknown
cHL14	37	Male	Caucasian	20/02/2014	Spleen	Metastatic	White	Nodular Sclerosing	Post Treatment	III

Supplementary Table 1: cHL patients clinical information

Supplementary Figures

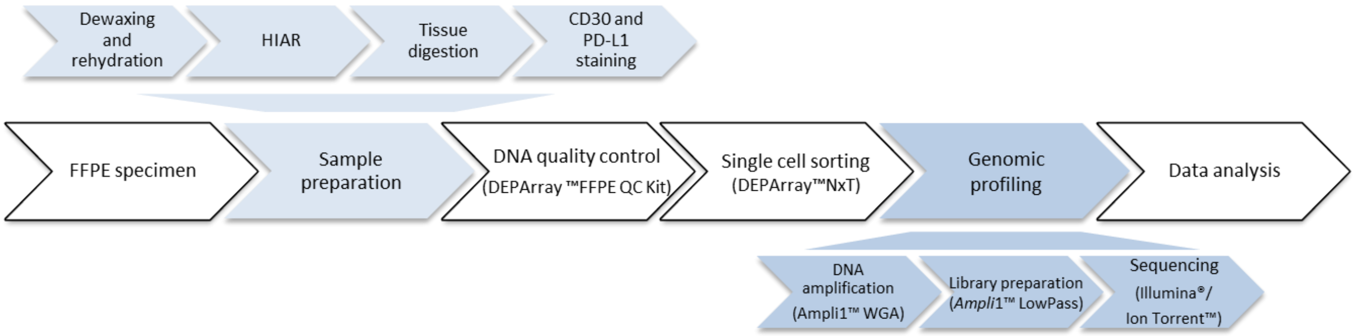

Supplementary Figure 1: Experimental step-by-step workflow: One section of a formalin-fixed, paraffin-embedded tissue block was processed as described in the subsidiary light-blue diagram above the mainstream process. The section was dewaxed and then rehydrated by sequential incubations in decreasing grades of alcohol solution. Antigens were unmasked using a Heat Induced Antigen Retrieval (HIAR) pH 8.0 solution. In order to obtain a cell suspension the tissue was subjected to a mechanical/enzymatic dissociation. Cell suspension was then stained using anti-CD30 and anti-PD-L1 antibodies. After sample preparation, the quality of DNA was assessed and DEPArray™ NxT system was used to isolate single HRS cells and leukocytes used as a control. After recovery, target cells were whole genome amplified and genome-wide copy-number alterations (CNAs) profiles were obtained. The steps for genomic profiling are listed in the blue diagram below the mainstream process.

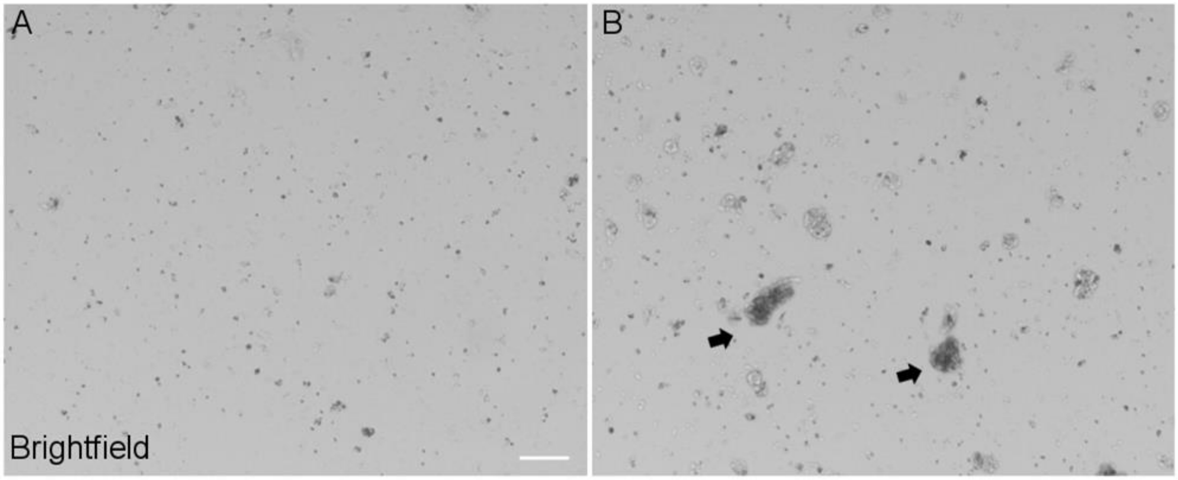

Supplementary Figure 2: Dissociation step monitoring. A) cHL sample properly dissociated. B) cHL sample with undissociated clusters (highlighted with black arrows).

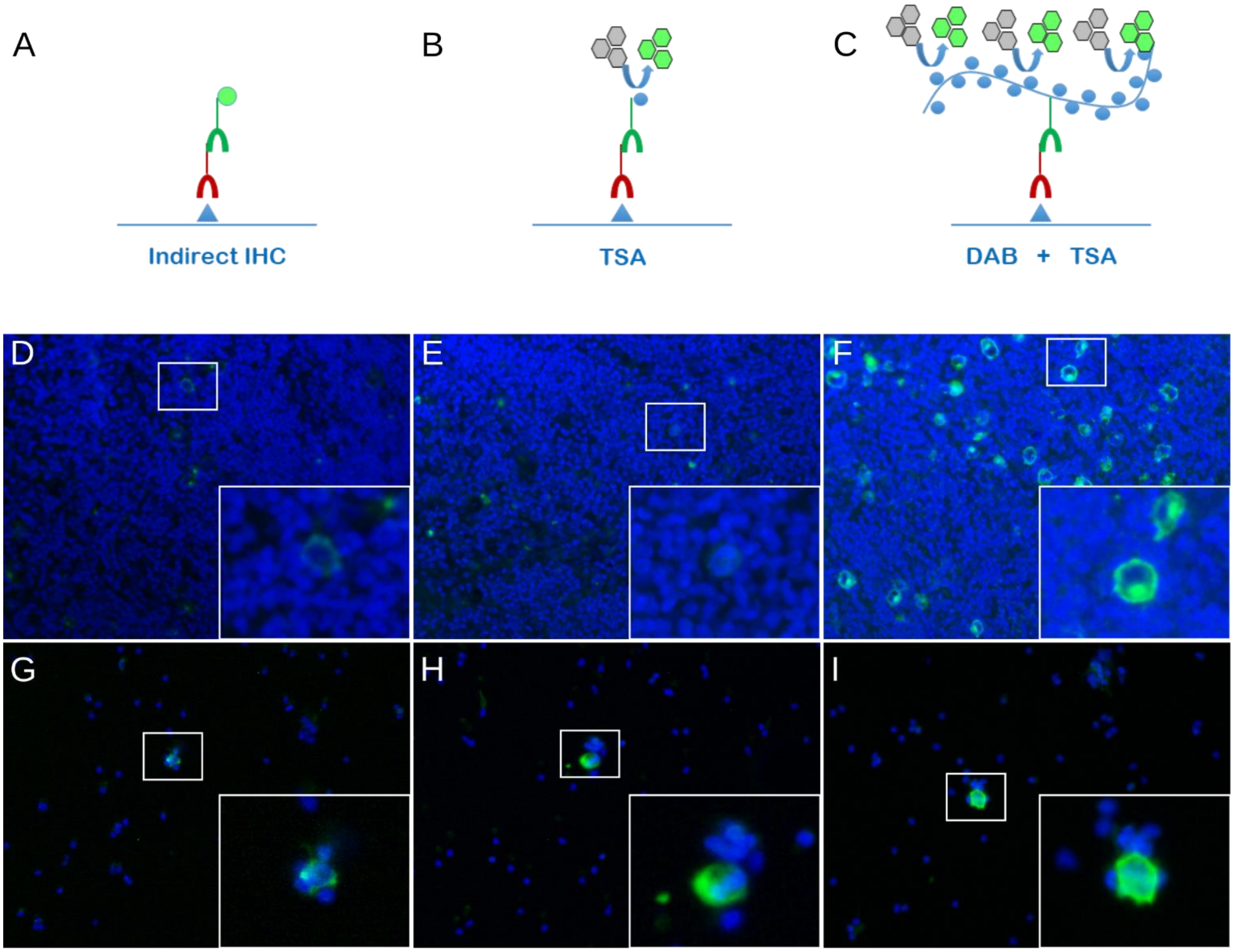

Supplementary Figure 3: Set-up of CD30 staining. CD30 staining was set-up both on microscope slides and on dissociated cell suspension. Indirect immunohistochemistry (IHC) using as detection system a fluorescent dye-conjugated secondary antibody (A) was compared to immunoenzymatic assays designed to amplify the signal generated by the enzyme horseradish peroxidase (HRP). In the tyramide signal amplification (TSA) immunoenzymatic assay, HRP is directly conjugated to a secondary antibody (B), while in the DAB immunoenzymatic assay, numerous HRP molecules are conjugated on a backbone of dextran polymer conjugated to a secondary antibody (C). Alexa Fluor™ 488 Tyramide Reagent was used as HRP substrate. D), E) and F) Representative images of microscope slides of a sample stained using indirect IHC, TSA, and DAB+TSA, respectively. G), H) and I) Representative images of cell suspension obtained from a sample stained using indirect IHC, TSA, and DAB+TSA, respectively.

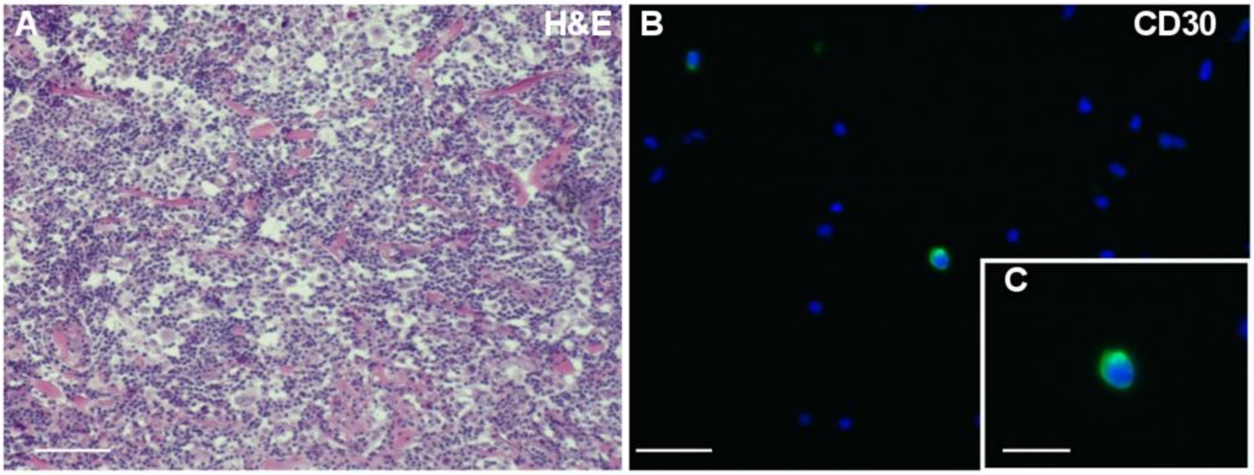

Supplementary Figure 4: Sample preparation for HRS single cell identification and purification. A) Representative haematoxylin and eosin (H&E) micrograph of a lymph node section of a cHL patient (scale bar: 100 µm). B) Cell suspension stained for CD30 (in green) and DAPI (in blue), obtained from a lymph node FFPE section using the sample preparation workflow (scale bar: 100µm) . C) Magnification of a HRS cell (scale bar: 50 µm).

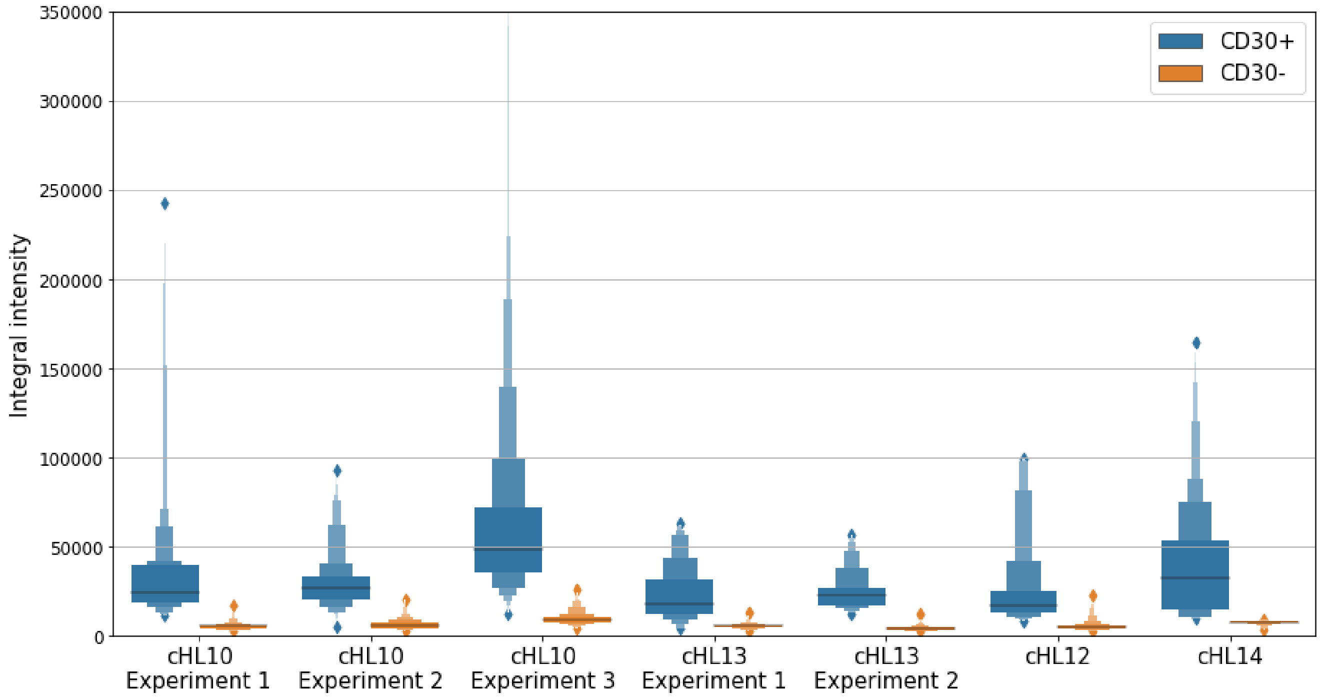

Supplementary Figure 5: Distribution of PD-L1 fluorescence channel integral intensity. For all samples a clear difference of PD-L1 channel signal has been observed between CD30- and CD30+ cell populations.

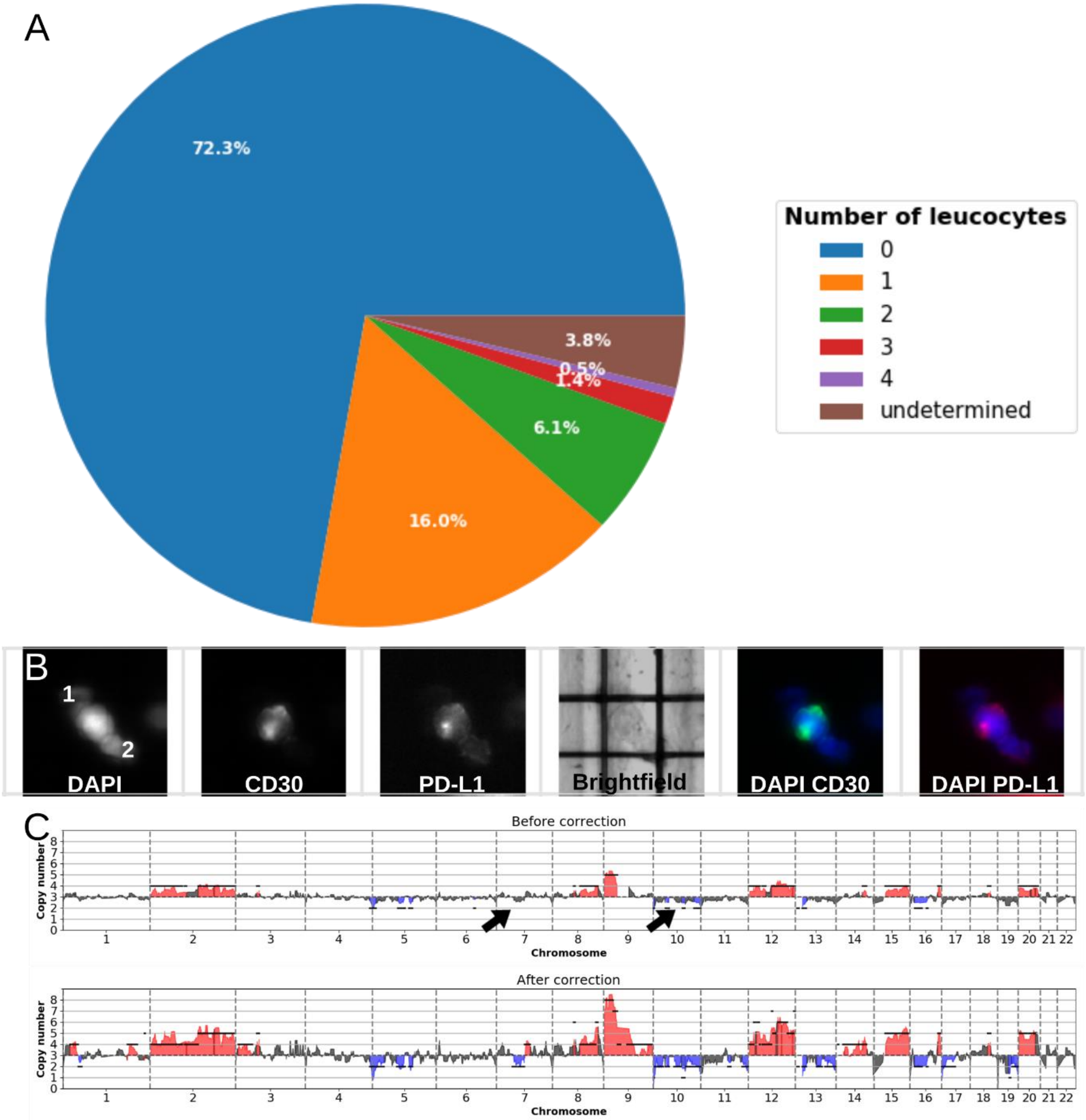

Supplementary Figure 6: Correction of copy number profiles based on imaging of single cells. Following method optimization, a small percentage of CD30+ cells, still retains rosetting leukocytes after dissociation: this is in line with several studies describing the presence of rosetting T cells trying to eradicate the disease but failing to recognize and eliminate tumor cells due to several immune evasion mechanisms. Thanks to the image-based selection adopted in this workflow it is possible to preferentially select single HRS cells or, in presence of up to 4 rosetting leukocytes, to correct the copy number profile according to the numer of contaminating cells. A) Distribution of contaminant leucocytes in single CD30+ cell recoveries. B) Image gallery of a HRS rosetted by two leukocytes (1 & 2). C) Copy number profiles have been corrected based on the number of leukocytes surrounding the HRS improving the sensitivity of the assay and allowing a more accurate determination of absolute copy number changes. Black arrows indicate copy number alterations whose signal is compressed and does not match integer copy number values before correction. After correction the signal of the same alterations matches integer copy number levels with high precision.

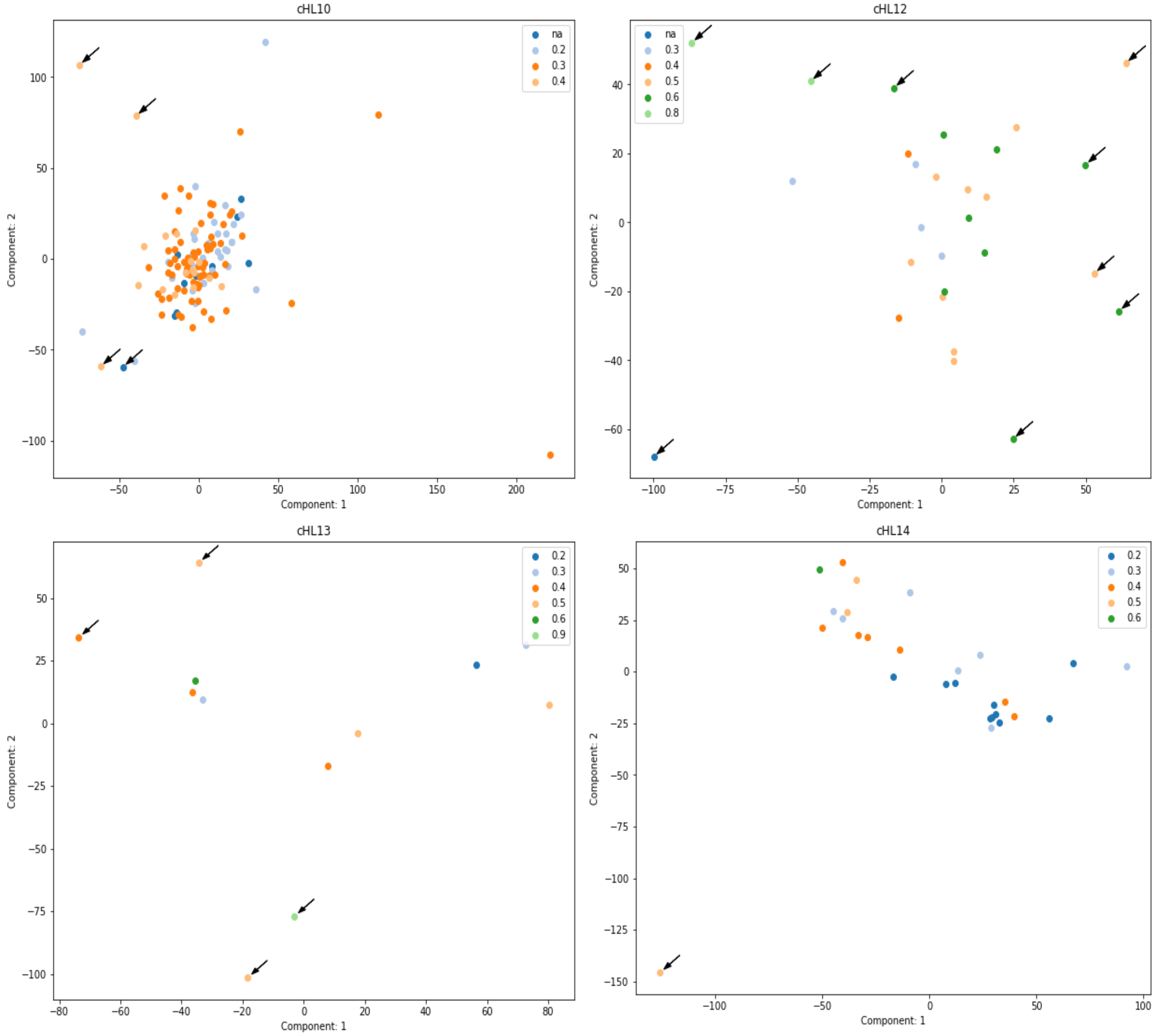

Supplementary Figure 7: PCA analysis of copy number alteration profiles for each sample. First two components are plotted one against the other. Dots are colored depeding on derivative log ratio spread values. Profiles were considered as putative outliers when they do not cluster near other samples on the PCA plot and DLRS >= 0.4 (black arrows).

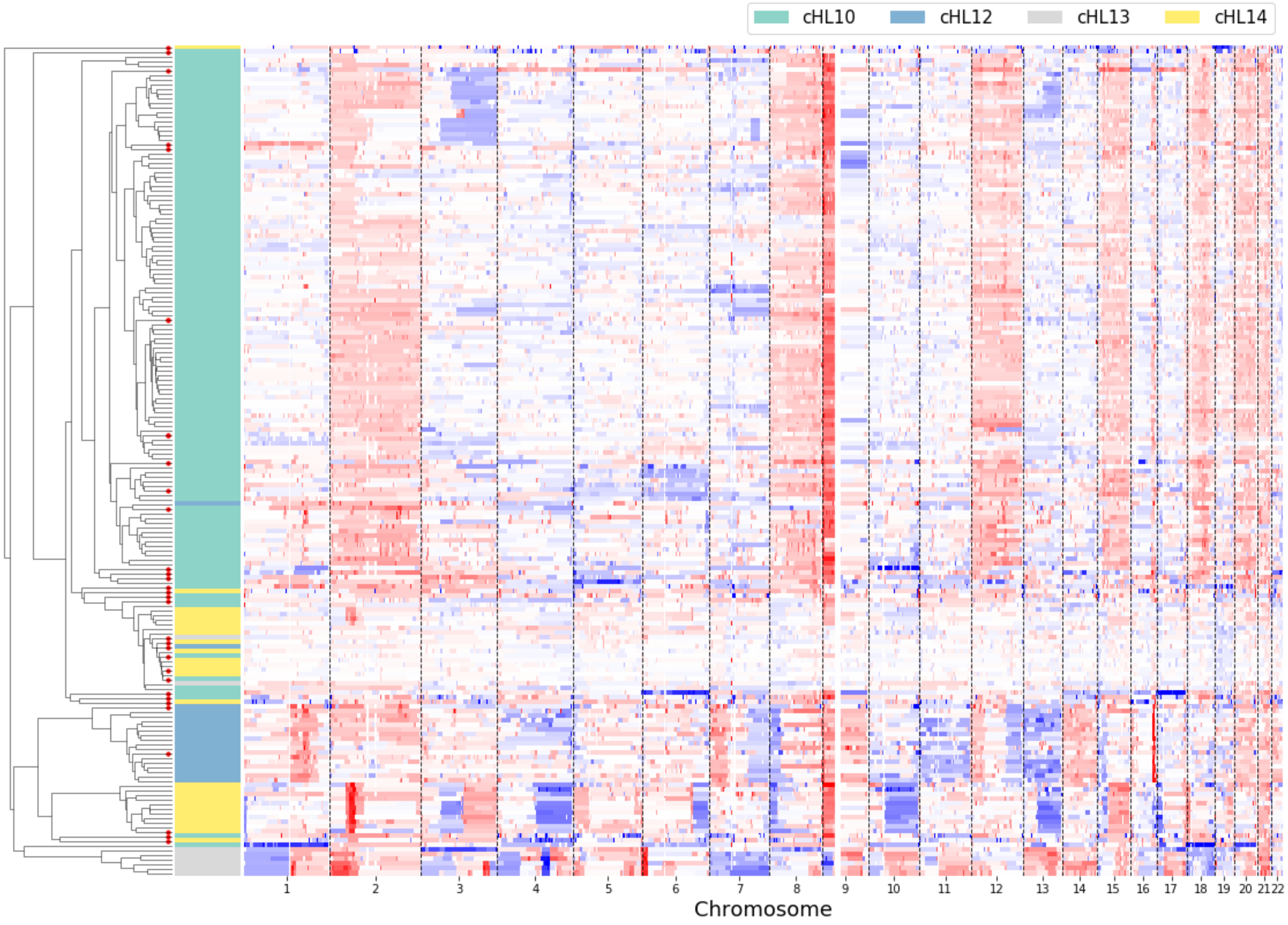

Supplementary Figure 8: Hierarchical clustering of whole genome copy number profiles. Clustering was performed using euclidean distance and Ward method. Rows correspond to samples, while columns correspond to genomic positions along the 22 autosomes. Samples contaminated from leukocytes whose profile did not cluster along with other cells from the same patient or whose profile was dissimilar from those of the other cells were excluded from further analyses (red dots).

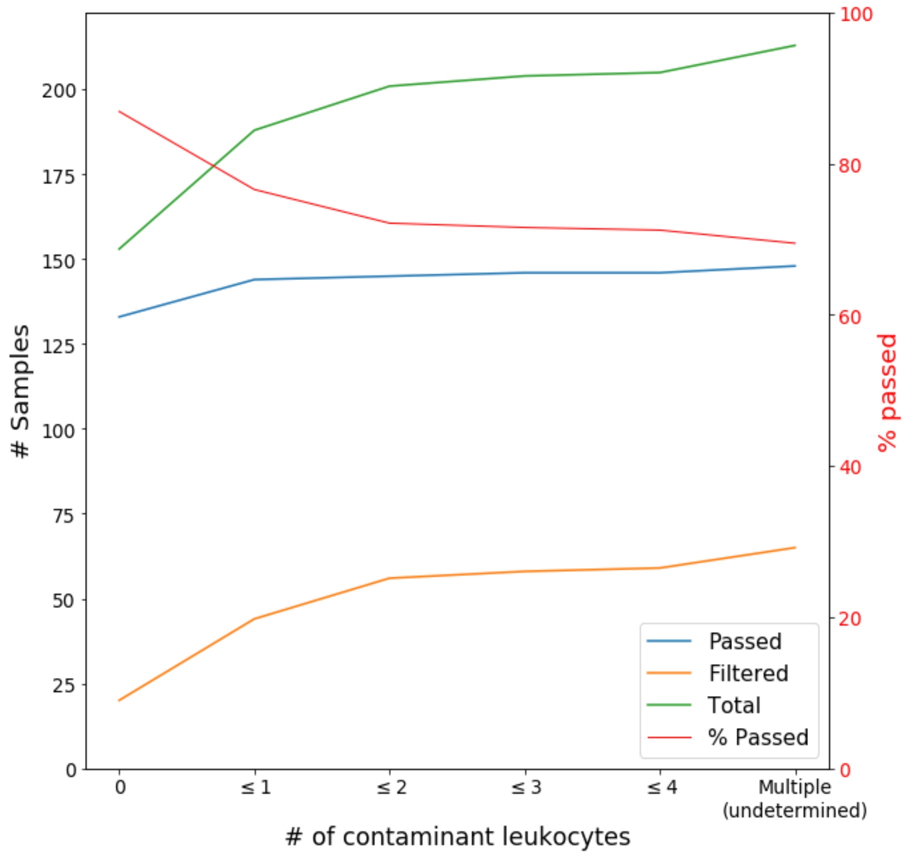

Supplementary Figure 9: Whole genome copy number data filtering statistics at increasing number of contaminant leukocytes. Most (86%) of whole genome copy number profiles from pure single CD30+ cells are retained after filtering. About one third of copy number profiles single cells contaminated by a single leucocyte could be effectively corrected and passed the filtering steps.

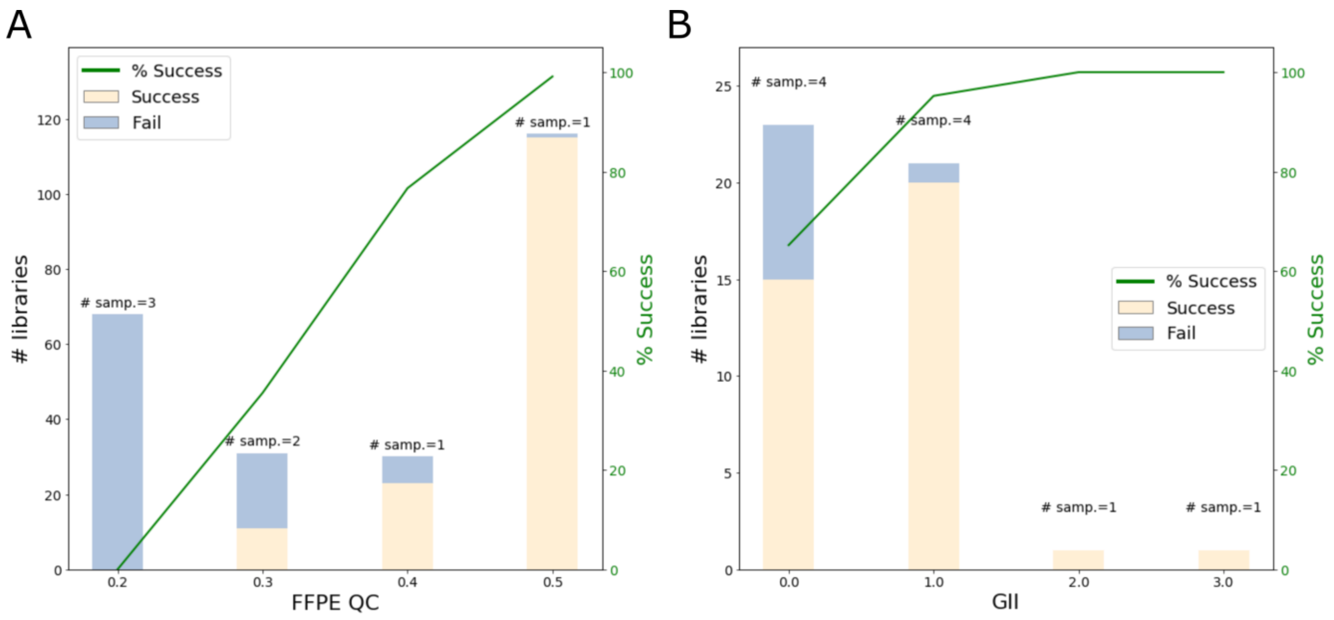

Supplementary Figure 10: Library success rate. In A) is shown the single CD30+ cell library success rate as a function of DEPArray™ FFPE QC score and in B) the library success rate as a function of WGA Genome Integrity Index (GII). On the X axis is the score, while on the left Y axis is the number of libraries and on the right Y axis is the percentage of libraries defined as successful. On top of each bar is shown the number of FFPE biopsy samples tested. A library was defined as successful when DLRS of the copy number profile obtained was lower than 0.4. FFPE QC score showed a high correlation with success rate and is thus suitable as a preliminary quality control to qualify DNA for single-cell workflow. In case of GII we tested a subset of WGA products and a large number of them showed a low GII, comprised between 0 and 1; this is expected given the high DNA fragmentation typical of FFPE samples. Anyhow, a large percentage (>60%) of single-cell with GII=0 produced high quality data (DLRS <0.4). Thus, WGA GII is not predictive of final library quality and is not suitable as quality control for single FFPE cells.
